# Predictors for patient knowledge and reported behaviour regarding driving under the influence of medicines: a multi-country survey

**DOI:** 10.1186/1471-2458-12-59

**Published:** 2012-01-20

**Authors:** Susana P Monteiro, Liset van Dijk, Alain G Verstraete, F Javier Álvarez, Michael Heissing, Johan J de Gier

**Affiliations:** 1 Department of Pharmacotherapy and Pharmaceutical Care, University of Groningen, Antonius Deusinglaan 1 9713AV Groningen, The Netherlands; 2 Driving Under the Influence of Drugs, Alcohol and Medicines (DRUID) project, Rue de la Loi/Wetstraat 175, BE-1048 Bruxelles, Belgium; 3 NIVEL, Netherlands Institute for Health Services Research, PO Box 1568, 3500, BN Utrecht, The Netherlands; 4 Department of Clinical Chemistry, Microbiology and Immunology, Ghent University, De Pintelaan 185, 9000 Ghent, Belgium; 5 Department of Pharmacology and Therapeutics. Faculty of Medicine, University of Valladolid, 47005 Valladolid, Spain; 6 BASt, Federal Highway Research Institute, Brüderstraße 53, 51427 Bergisch Gladbach, Federal Republic of Germany

## Abstract

**Background:**

Reports on the state of knowledge about medicines and driving showed an increased concern about the role that the use of medicines might play in car crashes. Much of patient knowledge regarding medicines comes from communications with healthcare professionals. This study, part of the DRUID (Driving Under the Influence of Drugs, alcohol and medicines) project, was carried out in four European countries and attempts to define predictors for knowledge of patients who use driving-impairing medicines. The influence of socio-demographic variables on patient knowledge was investigated as well as the influence of socio-demographic factors, knowledge and attitudes on patients' reported behaviour regarding driving under the influence of medicines.

**Methods:**

Pharmacists handed out questionnaires to patients who met the inclusion criteria: 1) prevalent user of benzodiazepines, antidepressants or first generation antihistamines for systemic use; 2) age between 18 and 75 years old and 3) actual driver of a motorised vehicle. Factors affecting knowledge and reported behaviour towards driving-impairing medicines were analysed by means of multiple linear regression analysis and multiple logistic regression analysis, respectively.

**Results:**

A total of 633 questionnaires (out of 3.607 that were distributed to patients) were analysed. Patient knowledge regarding driving under the influence of medicines is better in younger and higher educated patients. Information provided to or accessed by patients does not influence knowledge. Patients who experienced side effects and who have a negative attitude towards driving under the influence of impairing medicines are more prone to change their driving frequency behaviour than those who use their motorised vehicles on a daily basis or those who use anti-allergic medicines.

**Conclusions:**

Changes in driving behaviour can be predicted by negative attitudes towards driving under the influence of medicines but not by patients' knowledge regarding driving under the influence of medicines. Future research should not only focus on information campaigns for patients but also for healthcare providers as this might contribute to improve communications with patients regarding the risks of driving under the influence of medicines.

## Background

It has been known for many years that the consumption of psychoactive substances, such as alcohol, sedatives, anxiolytics, antidepressants or illicit drugs, has a negative effect on the ability to drive [1]. In fact, either alone or in combination, alcohol and psychoactive substances increase the risk of having a traffic accident [1-7]. According to the European Commission's Directorate-General for Mobility and Transport, Road Safety Unit, 25% of accidents involve alcohol, medicines, or illicit drugs. These accidents are directly responsible for the loss of 10.000 lives due to car crashes in Europe every year [8]. Worldwide, governments and authorities invest a great amount of money and effort in changing the behaviour of road users not only with respect to the use of seat belts and speed limits but also towards driving under the influence of alcohol, illicit drugs and medicines. Despite all the attempts and all the road safety campaigns that have been launched, traffic accidents are still responsible for more than 40,000 deaths and 1.7 million injuries across Europe [9].

Therefore, special efforts are needed in order to have a better knowledge of the various aspects of this specific problem and to develop appropriate solutions. This is the reason why an EU-project under the acronym of DRUID (Driving under the Influence of Drugs, Alcohol and Medicines) [10] was funded by the European Commission. DRUID aims not only to improve the possibilities of detecting drug-influenced driving in Europe but also to combat the scourge of drunk-driving and find answers to the question of the use of drugs or medicines that affect people's ability to drive safely

Reports on the state of knowledge about drugs and driving [1] showed an increased concern about the role that the use of medicines might play in traffic accidents. It has been estimated that 5-10% of medicines impair driving performance [2] as a consequence of their effects or side effects. This is the reason why it is so important to inform drivers who use driving-impairing medicines of the risks of driving under the influence. Research conducted in European countries has shown that patients do want to be informed about their medicines, their risks [11] and side-effects [11-13]. Similarly, in the U.S., two-thirds of patients reported the desire to be informed of all possible side effects of their medicines [14]. Much of patient knowledge regarding their medicines comes from communication with healthcare professionals, such as general practitioners and pharmacists, media exposure, and reading the safety information on the medicine's label [15]. Age and educational level seem to influence patient awareness and knowledge as health literacy decreases with increasing age [15].

In order to better understand and predict human behaviour, some theories have been derived to describe and explain how and why people behave the way they do. The theory of planned behaviour (TPB), the theory of interpersonal behaviour (TIB), and other more recent models with direct application in health research are examples of what has been done in the field of predictive behaviour. TBP was first suggested by Fishbein and Ajzen [16] and TIB is an extension of TBP. Both theories focus on intentions that are personal decisions to perform behaviour and are based on someone's knowledge about themselves and about the world around them. In TBP, intensions are based on attitudes towards the behaviour, subjective norms, and perceived behaviour control. These three aspects form an intention that, ultimately, leads to a behaviour. TIB, on the other hand, integrates normative and social factors into TBP. In this theory, perceived consequences of behaviour and habits are predictors of intentions that, like in the TBP, lead to behaviour. It might be true that influencing attitudes do not always result in a change of behaviour. Nevertheless, recent studies state that attitude is linked to traffic violations, especially when related to speed limits violations [17], use of seat belts [18], and driving under the influence of alcohol.

**Figure 1:**
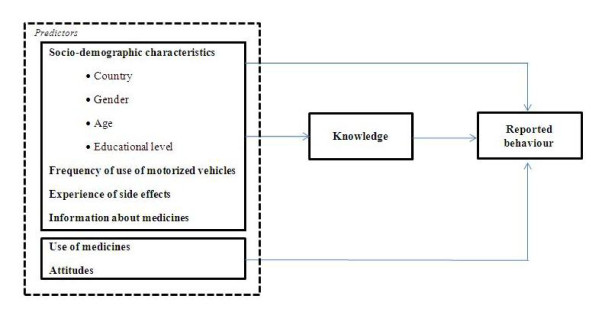
**Theoretical model to determine predictors for patient knowledge and reported behavior**.

Having the TBP and TIB as a main reference, this research attempts to determine predictors that can influence not only patients' reported behaviour, but also their knowledge. In a simplified way, the theoretical reasoning behind the construction of the models was based on the assumption that socio-demographic characteristics play a central role in knowledge (evaluated as knowledge about risk of having a traffic accident). Both socio-demographic characteristics and knowledge in combination with attitudes, defined as feelings towards driving under the influence of medicines, were used to predict reported behavior (in terms of change in driving frequency and/or in terms of change in the use of medicines). Figure 1 exemplifies the model that was developed based upon mentioned theoretical insights. The present study is part of DRUID, and it was conducted in 4 countries.

## Methods

### Design

The study was conducted in four countries: Belgium, Germany, Spain and the Netherlands, in 2007. The reason why these four countries were selected is because the Ministry of Transport or the national organization of pharmacists developed materials related to drugs and driving, and, therefore, they were very sensitive to the topic and, as a consequence, more willing to participate.

The questionnaire used in our study was derived from a previous Dutch national survey [19]. The original questionnaire was constructed by a multi-disciplinary team of experts and tested in over a 1000 consumers and patients.

Patients received the questionnaire through their pharmacy. Pharmacists were asked to hand out a questionnaire to approximately 1.000 patients (per country) who went to a community pharmacy and met the established inclusion criteria (see below).

The study was approved by the Medical Ethical Committee of the Universitair Medisch Centrum Groningen (University Medical Centre Groningen), the Netherlands as well as by the Medical Ethics Committee of the Universidad de Valladolid (Spain) and Ghent University Hospital Medical Ethics Committee (Belgium). Patient anonymity was ensured and all data were kept confidential.

### Pharmacies

Different approaches were used to select the pharmacies, depending on the country.

#### Belgium

In Belgium, only Flemish pharmacies were approached. A message was spread through the APB (Algemene Pharmaceutische Bond--Belgian Pharmaceutical Association) and Escapo (an authorised wholesaler) to a selected number of pharmacies (93 in total). A total of 40 pharmacies (43% response rate) agreed to participate in the study.

#### Germany

In collaboration with ABDA (Bundesvereinigung Deutscher Apothekerverbände--Federal Union of German Associations of Pharmacists), a total of 3.600 pharmacies, part of the ABDA network, were invited to participate in the study, from which 38 pharmacies (1% response rate) accepted the invitation.

#### The Netherlands

The pharmacies invited to participate in the Dutch patient questionnaires were selected among all the pharmacies that already cooperated with the University of Groningen, Pharmacy (RuG-Pha). Pharmacies that were participating in on-going projects were excluded. A total of 93 pharmacies were invited to participate in the study and 36 pharmacies (39% response rate) accepted the invitation.

#### Spain

In Spain, the institute responsible for the dissemination of the questionnaires was the University of Valladolid (UVa), in collaboration with the Spanish Society of Community pharmacies (Sociedad Espańola de Farmacia Comunitaria). Nationalwise, a total of 80 pharmacies were invited to participate. Of these, 35 (44% response rate) were actually enrolled in the study.

Pharmacists who agreed to participate in this study were not trained for this study. Yet, all participating pharmacists were provided with detailed information about the study, which included a list of participant inclusion criteria translated into the national language of each country. The reliability of the method to select patients was assured by addressing questions covering the inclusion criteria in the questionnaire and by excluding the patient from the analysis whenever the inclusion criteria was not fulfilled. Pharmacists looked for potential participants during working hours for a period of 3 weeks.

### Patient inclusion criteria

Patients were eligible to participate in the study if they met the following inclusion criteria:

1. The patient uses anyone of the following medicines that are known to impair driving ability:

2. The patient is a prevalent user (2nd prescription or more); ensuring that they experienced any possible side effects and resulting changes in reported behaviour.

3. The patient is 18-75 years of age, as it is known to be the age group that actively participates in driving and that takes medicines.

4. The patient actually drives a motorised vehicle in traffic (is to be asked at the time of dispensing).

• Benzodiazepines (as *hypnotic or anxiolytic*): flunitrazepam, flurazepam, loprazolam, nitrazepam, zopiclon, alprazolam, bromazepam, buspirone (> 2dd 10 mg), chlordiazepoxide, diazepam, lorazepam, oxazepam.

• Antihistamines (*1st generation antihistamines*): clemastine, dexchlorpheniramine, promethazine, cyproheptadine, tripelennamine, hydroxyzine.

• Antihistamines (*1st generation antihistamines*): clemastine, dexchlorpheniramine, promethazine, cyproheptadine, tripelennamine, hydroxyzine.

When the patient met the four criteria mentioned above, he/she was asked to participate in the survey. A patient information letter, a paper questionnaire, and a stamped addressed return envelope were given to patients who agreed to participate in the study. Participants filled in the questionnaire at home and no information on the time for completion of the questionnaire was asked.

### Non-respondents

No records on the non-respondents were kept. This methodological decision was made for a practical reason based on the extra administrative work that pharmacists would have to do, which would have drastically decreased the number of pharmacists enrolled in the study. As a consequence, it is not possible to correlate participants' refusal patterns with time consumed filling in the questionnaire, age, gender, or any other socio-demographic variable.

### Questionnaire

The questionnaire was developed in English and, after consensus about the content, it was translated into the national language of each participant country (it was decided to use only a Dutch version for Belgium). Thereafter, reverse translation back into English was performed to ensure consistency between the translations.

The questionnaire consisted of 43 questions (39 closed questions and 4 open questions). The questions were grouped into 6 topics of interest covering general information (including information related with the inclusion criteria), participation in traffic, medicines in traffic, use of medicines, information about medicines, and attitude towards reported behaviour in traffic. By doing so, it was possible to collect data on general items regarding the patient characteristics as a participant in traffic and about patients medicine use. Specific items to assess patient knowledge, attitude, and reported behaviour were gathered as well. The questionnaire was provided as an Additional file 1 to the journal and can be find online. While pre-testing the questionnaire, it was verified that it would take between 15 and 20 min to be completely filled in.

### Dependent variables construction

#### Knowledge

Patient knowledge regarding medicines that have a hazardous influence on driving ability was assessed with respect to the knowledge about the risk of having a traffic accident when driving under the influence of driving-impairing medicines. By using a 5-scale parameter (1 = totally disagree and 5 = totally agree), patients could agree (or disagree) with the following sentences: "the risk of having a road accident is smaller when you have just started taking a driving-impairing medicine compared to long term treatment" or "the risk of having a road accident is similar when you take more of a driving-impairing medicine than prescribed" or "the risk of having a road accident remains the same when you use several driving-impairing medicines at the same time." The internal consistency of the scale was checked by means of the Cronbach alfa coefficient (0.64) and the sum score was calculated.

#### Reported behaviour

Patient reported behaviour was analysed in terms of a change in their frequency of driving (yes or no) or in terms of changing the use of driving-impairing medicines (yes or no). For each questions, the sum score of the different statements was calculated. Table 1 shows the answers participants provided, as well as the number of participants that answered each question.

**Table 1:** Statements on changes in reported behaviour, concerning driving ability and use of driving-impairing medicines

	Frequency (4 countries)
*Statements on changes in frequency of driving*	*N = 305*

Participants decided not to change frequency of driving because:	

• "I did not think the information was relevant to me";	25

• "It was not feasible for me to change my frequency of driving";	41

• "I did not notice any negative effects that influenced my driving ability";	108

• "I found information stating that the medicine does not have any driving-impairing effects";	32

• "other reasons".	19

Participants decided to change frequency of driving because:	

• "I decided not to drive my motorised vehicle anymore";	17

• "I decided to drive/ride a motorised vehicle less often";	26

• "I decided to drive/ride a motorised vehicle on less parts of the day" (e.g. only during day light);	19

• "other reasons".	18

*Statements on changes in the use of driving-impairing medicines*	*N = 296*

Participants decided not to change the use of driving-impairing medicines because:	

• "I did not think the information was relevant to me";	78

• "There was no alternative medicine available";	62

• "other reasons".	46

Participants decided to change the use of driving-impairing medicines because:	

• "I decided not to use the medicine";	4

• "I decided to use (most of) the medicine at night instead of during the day";	62

• "I decided to only use the medicine when I did not need to drive";	24

• "I asked for or I was prescribed a medicine causing less impairment of the ability to drive".	8

• "other reasons".	12

### Independent variables construction

The following variables were included in the multi- and logistic- regression analysis:

• Country (Belgium, Germany, the Netherlands and Spain).

• Patient gender and age category (18-25; 26-34; 35-44; 45-54; 55-64; 65-75. Age category 55-64 was used as reference as it is the age group that takes more medicines).

• Educational level (not completed primary school, completed primary school, lower vocational training, intermediate vocational training and university degree entered as dummy variables in the analysis. Intermediate vocational training was used as reference).

• Use of motorised vehicles (participants were considered as "frequent users" when they used their motorised vehicle "5-7 days per week" or "2-4 times per week"; "sporadic users" were the ones using a motorised vehicle "2-4 times per month" or "once a month or less" or "never." The scale to assess frequency of driving was previously used in other studies in this same field [20]).

• Experienced side effects that can potentially impair driving ability such as sleepiness or drowsiness, decrease of alertness, problems concentrating, blurred vision and dizziness (yes/no).

• Information received from healthcare providers related with the influence of medicines on driving ability (yes/no).

• Information looked up by patients themselves, related to the influence of medicines on driving ability (yes/no).

• Whether patients read the Patient Information Leaflet (PIL) or not (yes/no). This question did not attempt to investigate comprehension. Instead, it was used to know whether patients used the PIL as source of information.

• Patient attitude-In order to assess patient attitudes towards the use of medicines that potentially impair patients while driving, a 5-scale parameter (1 = totally disagree and 5 = totally agree) was used to analyse patient's opinion in relation with the use of impairing medicines and driving as well as the consequences of driving under the influence of impairing medicines. In both cases, the internal consistency of the scale was checked by means of the Cronbach alfa coefficient (0.85 and 0.69 respectively) and the sum score was calculated. To investigate patient attitude towards the use of impairing medicines, questions such as "when I drive while using driving-impairing medicines I endanger my personal safety" or "when I have been prescribed a driving-impairing medicine I choose not to use my car and choose other types of transportation" or "when I have been prescribed a driving-impairing medicine, I try to use my car as little as possible" were asked. Questions such as "the risk of driving under the influence of driving-impairing medicines is being exaggerated" were used to address patient attitude towards the consequences of driving under the influence of impairing medicines.

### Data-analysis

Guidelines were developed for all participating countries in order to ensure that data entry was conducted the same way. Additionally, all databases were checked for undefined values and extreme outliers. Data were analysed using SPSS 16.0 for Windows.

Descriptive analysis was used to report on respondent characteristics such as gender, age and educational level. Multiple linear regression analysis was used to identify patient knowledge about driving while using medicines that might impair driving ability, whereas reported behaviour was analysed by means of multiple logistic regression. Multiple linear regression analysis could not be used in both situations because this statistical test is only applicable when the dependent variable is continuous (which is the case with investigating participant knowledge). As behaviour is a dichotomous variable (yes/no), multiple logistical regression was applied in order to predict which factors influence patient behaviour. Knowledge and reported behaviour were considered as dependent variables of the equation. Independent variables, those that can theoretically influence the dependent variables, consisted of country, gender, age category, educational level, use of motorised vehicles, self-report of experienced side effects, information about medicines (either received from healthcare providers, looked up by patients themselves, or stated in the PIL), intake of medicines, knowledge itself, and, finally, attitudes towards use of medicines and driving and towards driving under the influence. Prior to the construction of the models, univariate analysis of variance was conducted to verify whether or not there was an interaction effect between the consumption of medicines and age influencing patients' knowledge. As no interaction effects between medicines' consumption and age were found, it was decided not to include them, as variables, in the models. A *p-value *< 0.05 was considered as statistically significant.

## Results

Data collected from 633 questionnaires (overall response rate of 18%) were used, as shown in Table 2. Ninety-three questionnaires that were returned could not be used for the analysis for several reasons: questionnaires came back with no answers (54.6%); questionnaires belonging to patients that did not meet the inclusion criteria (36.4%); and questionnaires with no reference to the medicines that participants were taking and, simultaneously, patients stated not taking any medicine (3.3%).

**Table 2:** Number of responses per country

Country	Number of questionnaires sent out to pharmacies	Number of questionnaires returned	Response rate (%)	Number of questionnaires used in the analysis (after screening the inclusion criteria)
Belgium	1000	144	14.4	136

Germany	880	162	18.4	146

The Netherlands	830	150	18.1	136

Spain	960	270	28.1	215

*Total*	3670	726	17.75 (mean)	**633**

The relevant characteristics of the study population are summarised in Table 3. The majority of the respondents were female and almost 50% of the total population was between 35 and 54 years old. Regarding educational level, there is a slightly higher number of patients with a university degree (27.6%) compared to other levels of education. Another relevant characteristic of participants is how often they use motorised vehicles. Participants of our study were mainly frequent users of motorised vehicles, meaning they used their car on a daily or weekly basis.

**Table 3:** Characteristics of the respondents, per country

	Country								p-value*
	
	Belgium		Germany		Netherlands		Spain		
	
*Total number of participants*	136		146		136		215		
	
	n	%^a^	n	%^a^	n	%^a^	n	%^a^	
**Gender**									

Male	135	34.6	145	34.2	136	41.2	214	47.0	0.175
									
Female		64.7		65.1		58.8		52.6	

**Age Category**									

18-25		5.9		1.4		3.7		6.5	
									
26-34	134	11.8	145	10.3	136	5.9	213	20.5	< 0.001*
									
35-44		18.4		24.7		26.5		27.0	
									
45-54		32.4		21.2		23.5		22.8	
									
55-64		15.4		19.2		27.2		14.0	
									
65-75		14.7		22.6		13.2		8.4	

**Educational level**									

Not completed primary education		11.0		0.7		1.5		5.1	
									
Completed primary education		14.7		32.2		5.9		19.1	
									
Lower vocational training	132	22.8	145	32.2	128	19.1	215	16.7	< 0.001*
									
Intermediate vocational training		25.7		17.8		41.9		19.5	
									
University		22.8		16.4		25.7		39.5	

**Use of motorised vehicles**									

Sporadic users	136	33.1	146	34.9	136	47.8	215	31.2	0.012*
									
Frequent users		66.9		65.1		52.2		68.8	

**Use of driving-impairing medicines**									

Use of 1 medicine		65.2		70.8		71.2		75.0	
									
Use of 2 medicines		28.1		25.0		23.5		21.2	
									
Use of 3 medicines	135	5.9	144	4.2	132	4.5	212	2.8	0.734
									
Use of 4 medicines		0.7		0.0		0.8		0.9	

**Experienced side effects**									

No	132	51.5	146	48.6	134	37.3	211	29.9	< 0.001*
									
Yes		48.5		51.4		62.7		70.1	

**Knowledge**^**b**^									

About the risk of having a road accident when using medicines^c^	2.4 (1.0)		2.4 (1.1)		2.3 (1.0)		2.3 (0.9)		0.882

**Attitude**^**b**^									
Use of medicines and driving^c^	2.3 (0.9)		2.8 (1.4)		1.9 (0.8)		2.1 (1.1)		< 0.001*

Consequences of driving under the influence of impairing medicines^d^	3.5 (0.9)		4.0 (0.9)		3.4 (0.9)		3.6 (0.8)		< 0.001*

**Reported behaviour **- change in the frequency of driving									

No	42	61.9	64	71.9	79	81.0	120	74.2	0.149
									
Yes		38.1		28.1		19.0		25.8	

**Reported behaviour **- change in the use of driving-impairing medicines									

No	38	57.9	65	55.4	75	81.3	118	56.8	0.002*
									
Yes		42.1		44.6		18.7		43.2	

^a^The percentage refers to within the country

^b^Knowledge and attitude results presented in terms of mean (standard deviation)

^c^5-item scale (1 = totally disagree; 2 = disagree; 3 = no opinion; 4 = agree; 5 = totally agree). Totally disagree means that the patient does not perceive any risk of driving under the influence of medicines

^d^5-item scale (1 = totally disagree; 2 = disagree; 3 = no opinion; 4 = agree; 5 = totally agree). Totally disagree means that patients have a negative opinion on the influence of medicines on driving performance and about the consequences of that behaviour

* *p*-value < 0.05 considered to be statistically significant. *P*-value was calculated by means of the Chi-square test.

**Figure 2:**
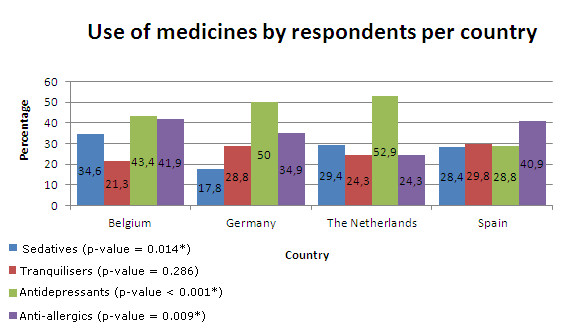
**Self-reported use of medicines by respondents per country (percentage within country)**.

Concerning self-reported medication that patients were taking, within countries, antidepressants were the most stated medicines (42.0%; n = 266) followed by anti-allergics (36.2%; n = 229), sedatives (27.5%; n = 174), and tranquilisers (26.5%; n = 168). Figure 2 shows the percentage of self-reported use of medicines. Antidepressants are the most frequently used group of medicines in each country except in Spain, where there is a higher percentage of anti-allergics and where there is a homogenous prevalence of consumption of sedatives, tranquilisers, and antidepressants. In the Netherlands, the prevalence of use of anti-allergics is the lowest. Only in Belgium is the trend of use of medicines the same as self-reported medication. An average of medication use in the 4 countries shows that patients took 1.32 medicines. 71.1% (n = 443) of the patients stated that they were taking only one medicine category at a time, and 0.6% (n = 4) of the patients combined all four medicine categories.

With regard to side effects, 40.4% (n = 252, Table 3) of patients stated that they did not experience any type of adverse effect. Only in Germany the percentage of patients who experienced side effects was higher than those who did not. From those who experienced side effects, "sleepiness" (24.1%) was most often stated, followed by "decrease in alertness" (12.7%), "problems concentrating" (10.6%), "dizziness" (5.8%), and "blurred vision" (4.2%). For all studied medicines, relevant side-effects were all listed in the PIL, which is accessible to patients and was read entirely by 55.8% (n = 348) of the respondents; 30.6% (n = 191) of patients read only parts of the PIL.

In Belgium, the percentage of patients stating they were not informed was higher, though not significantly than any other country. 63.6% (n = 84) of Belgian participants did not receive this information. Considering the four countries together, 62.5% (n = 349) of the patients received information from healthcare providers.

Patient knowledge about the risk of having a road accident was similarly low in all countries (Belgium and Germany 2.4 and the Netherlands and Spain 2.3 on average on a 5-item scale, where 5 is the highest score). Regarding reported behaviour (Table 3), 26.2% of patients were in favour of a change in driving frequency, while 37.2% were in favour of a change in the use of driving-impairing medicines. In terms of attitude towards the use of medicines while driving, patients disagreed with the statements that were presented in Belgium (average of 2.3), in the Netherlands (average of 1.9) and in Spain (average of 2.1); in Germany, patients did not have an opinion (average of 2.8). With respect to the attitudes towards driving under the influence of medicines, only German patients agreed (average of 4.0) with the fact that the risk of driving under the influence of medicines was exaggerated.

### Knowledge

Multiple linear regression analysis was used to assess factors influencing patient knowledge (Table 4) towards driving-impairing medicines. As previously mentioned, knowledge was considered in terms of risk of having a road accident. From Table 4, it is clear that independent variables explain 10.6% (adjusted R^2^) of the differences found between patients. The age of the patient is correlated with the knowledge about the risk of being involved in a traffic accident: the younger the patient the greater the knowledge about the risk. Regarding educational level, knowledge is higher among patients with an academic degree. Information patients received from healthcare providers, looked-up by patients themselves, or the information stated in the PIL did not appear to contribute to patient knowledge. The use of motorised vehicles had no effect on patient knowledge. Self-report of experience of side effects was also not significant.

**Table 4:** Predictors for patient knowledge on risks of having a road accident: multiple linear regression analysis

	Risk of having road accidents *(N = 617)*
**Independent variables**	**Standardised coefficients (*p*-value)**

Constant	3.469

**Country **(Germany used as reference)	-

Belgium	0.02 (0.798)

The Netherlands	0.00 (0.982)

Spain	-0.04 (0.529)

**Gender **(0 = male; 1 = female)	-0.03 (0.487)

**Age categories **(category 55-64 used as reference)	-

18-25	0.07 (0.134)

26-34	0.10 (0.048)*

35-44	0.16 (0.005)*

45-54	0.09 (0.092)

65-75	-0.13 (0.010)*

**Educational level **(Intermediate vocational training used as reference)	-

Not completed primary school	-0.14 (0.002)*

Completed primary school	-0.03 (0.621)

Lower vocational training	-0.05 (0.297)

University	0.18 (< 0.001)*

**Use of motorised vehicles **(0 = sporadic users; 1 = frequent users)	-0.04 (0.330)

**Experienced side effects **(0 = no side effects; 1 = side effects)	0.05 (0.283)

**Information received from healthcare providers** (0 = no information was provided; 1 = information was provided)	0.00 (0.970)

**Information looked for patients** (0 = no information was looked for; 1 = information was looked for by patients)	0.00 (0.927)

**Information from the PIL** (0 = patients don't read the PIL; 1 = patients read the PIL)	0.01 (0.893)

**Adjusted R^2^**	0.106

**F**	4.477

***p-value *(model)**	< 0.001*

### Reported behaviour

Multiple logistic regression was performed to assess the impact of patient reported behaviour with respect to changes in frequency of driving and the use of driving-impairing medicines (Table 5). The independent variables explain 34.8% and 27.3% (adjusted R^2^) of the variance for changes in driving frequency and in the use of driving-impairing medicines, respectively. Patients were more likely to change their frequency of driving when they are between 26 and 34 years old, when they report side effects, and when they have a negative attitude towards driving under the influence of impairing medicines. In contrast, patients who drive frequently and those who take anti-allergics tend to change their driving frequency less often compared to users of tranquilisers (Table 5). Concerning the model that takes into account changes in the use of driving-impairing medicines, patients who reported having experienced side effects and those who have knowledge about having a traffic accident appeared to change the use of driving-impairing medicines more often than those who came from the Netherlands or those who take antidepressants (Table 5).

**Table 5:** Changes in driving frequency and in use of driving-impairing medicines: multiple logistic regression analysis

Independent variables	Change in driving frequency (n = 305)			Change in the use of driving-impairing medicines (n = 296)		
	
	*Odds-ratio*	*95% CI for OR*		*Odds-ratio*	*95% CI for OR*	
				
		*lower*	*upper*		*lower*	upper
**Country **(Germany used as reference)						

Belgium	2.00	0.53	6.41	0.79	0.29	2.19

The Netherlands	0.69	0.21	2.26	0.24*	0.08	0.69

Spain	0.63	0.23	1.72	0.46	0.20	1.07

**Gender **(0 = male; 1 = female)	1.70	0.97	3.00	1.18	0.77	1.80

**Age categories **(category 55-64 used as reference)						

18-25	4.87	0.93	25.49	0.81	0.18	3.52

26-34	4.17*	1.14	15.25	1.78	0.59	5.42

35-44	2.08	0.71	6.15	1.72	0.68	4.31

45-54	2.75	0.97	7.84	1.79	0.71	4.54

65-75	1.62	0.48	5.84	0.60	0.19	1.95

**Educational level **(Intermediate vocational training used as reference)						

Not completed primary school	1.14	0.17	7.65	0.76	0.14	4.15

Completed primary school	2.78	0.95	8.11	2.13	0.81	5.61

Lower vocational training	2.20	0.82	5.90	0.79	0.33	1.88

University	1.46	0.57	3.71	0.98	0.43	2.20

**Use of motorised vehicles**						

(0 = sporadic users; 1 = frequent users)	0.35*	0.18	0.69	0.58	0.31	1.10

**Experienced side effects**						

(0 = no side effects; 1 = side effects)	3.18*	1.40	7.21	3.39*	1.68	6.82

**Medicines **(Tranquilisers used as reference)						

Sedatives	0.77	0.36	1.62	1.07	0.55	2.08

Antidepressants	0.84	0.42	1.68	0.24*	0.12	0.50

Anti-allergics	0.24*	0.10	0.58	0.82	0.40	1.66

**Knowledge about the risk of having a road accident **(0 = totally disagree; 5 = totally agree)	0.932	0.65	1.33	1.49*	1.09	2.03

**Attitude**						

Use of medicines and driving (0 = totally disagree; 5 = totally agree)	0.88	0.64	1.20	1.08	0.82	1.43

Driving under the influence of driving-impairing medicines (0 = totally disagree; 5 = totally agree)	2.23*	1.43	3.50	1.29	0.91	1.85

**Adjusted R^2^**		**0.348**			**0.273**	

***p-value *(model)**	< 0.001*	< 0.001*				

## Discussion

The present survey showed that socio-demographic variables, such as age and educational level, influenced patient knowledge about causes of road accidents: younger and higher educated patients had better knowledge. Information that might have been provided to or accessed by patients contributed to patient knowledge regarding the risk of having a traffic accident. In contrast, patient reported behaviour did not appear to be influenced by any of the socio-demographic variables considered in the analysis. Instead, frequency of driving changed due to experience of side effects and attitudes towards driving under the influence of driving-impairing medicines. The use of motorised vehicles and intake of anti-allergic medicines had less influence on the frequency of driving. Regarding changes in the use of driving-impairing medicines, our model suggested that such changes were mainly influenced in a positive manner (meaning a change in reported behaviour) by experienced side effects and knowledge about the risk of having a road accident and negatively influenced by country (specifically The Netherlands) and by the use of anti-depressant medicines.

Among the 4 participating countries, self-reported medication use indicated that patients took, on average, 1.3 medicines that are known to be driving-impairing. For antihistamines [21] and antidepressants [22], a dose-dependent negative effect on driving was found in road-side tests previously conducted in the Netherlands. Nevertheless, patients still drive their car (or any other motorised vehicle) on a daily basis, which could be explained by the fact that they no longer experience side-effects. This is supported by previous research showing that an increased number of patients using psychotropic medicines regularly do not refrain from driving their car [23].

Sleepiness, decrease in alertness, problems concentrating, dizziness or blurred vision were reported at least one time during treatment by almost 60% of the participants. These side effects are presumed to be potentially dangerous for driving ability [15]. However, experiencing side effects does not seem to be enough to make patients quit driving. At best, experiencing side effects will change patients' frequency of driving. This could indicate that patients tend to underestimate the risk that is associated with driving under the influence of medicines [15] and that their knowledge about medicines' side effects is limited [14] or that the side effects occurred at other times than when they were driving. On the other hand, a fair percentage (40%) of patients did not report any side effects. This could be explained by the long term use of medicines (a requirement to be included in the study; see criteria inclusion section)., which may result in the development of a tolerance to the side effects [23], or by the relationship between medicine use and side effects, for example in Spain 70.1% of patients stated having experienced side effects, which can be related with the high prevalence (40.9%) of use of first generation anti-allergic medicines.

### Knowledge

Much of patients' knowledge regarding medicines and side effects, in general, comes from communication with their general practitioner [14,15]. Similar to the existing literature, the present study shows that the majority of the participants received information about the influence of their medicines on their ability to drive from healthcare providers (both general practitioners and community pharmacists). The only exception was seen in Belgium, where more than 60% of the participants stated not having been informed about medicines and driving. Several studies [24,25] concluded that the communications between clinicians and patients about the potential risk of medicines is often incomplete. If so, it is crucial to increase physician awareness of potentially driving-impairing medicines in order to improve the communication with patients who could benefit from better and more complete counselling.

The information that patients received from healthcare providers, or that patients looked up themselves, did not appear to be effective enough to influence patient knowledge regarding the risk of being involved in a traffic accident. This is in line with previous research where it is shown that patients who are at a greater risk of being involved in a traffic accident due to medicines do not necessarily have more knowledge of increased risk [15]. Age and educational level appear to be the only two variables capable of influencing patient knowledge about the risk of having a road accident. Younger patients and patients with a higher educational level seem to have a greater health literacy (knowledge) and, as a consequence, a bigger awareness of the danger of using medicines and driving than older patients and those with lower educational level. The theoretical model that was developed (Figure 1) is supported by the findings concerning knowledge, as socio-demographic characteristics had both a positive and negative impact on patient knowledge.

### Reported behaviour

Age is the variable with a greater positive impact in the model: participants between 26 and 34 years-old are the ones more likely to change their driving behaviour. Patient self-report of side effects also correlates positively with changes in frequency of driving; this means that patients who felt side effects are more likely to change their driving behaviour. The same holds true for attitude towards driving under the influence of medicines. This variable positively influences the model, meaning that patients with a stronger attitude towards the consequences of driving under the influence are the ones changing the frequency of driving. Conversely, the use of motorised vehicles influences patient reported behaviour negatively, i.e. the more patients drive the less they change their driving behaviour in terms of frequency of driving. The use of anti-allergic medicines also correlates negatively with the model, making patients taking these medicines less prone to change their frequency of driving. This could be due to the fact that patients might not be aware that anti-allergic medication has a negative effect on driving ability or that side-effects occur when the patient is not driving. Another explanation for this fact could be due to the less sedative effect of the 2nd generation antihistamines, and, therefore, patients do not recognize any signs of impairment on their psychomotor abilities and, as a consequence, patients feel safe to drive.

Living in the Netherlands, experiencing side effects, using antidepressants, and knowledge about the risk of having a traffic accident are the factors that significantly account for changes in the use of medicines. Patients living in the Netherlands and using antidepressant medicines change the use of medicines less often. This could be due to the fact that antidepressants are well known to affect driving. As a consequence, healthcare providers, such as physicians or pharmacists, can immediately provide patients with preventive measures (for example, taking the medicines at night), which might reduce the negative effects of the medicines on driving ability resulting in a less need to change driving frequency.

In contrast, self-report of experiencing side effects and knowledge about risk of being involved in a road accident contribute positively to change in the use of driving-impairing medicines. This means the more side effects a patient experiences and the greater their knowledge about the side effect, the bigger the chances that they will change the intake of their medicines.

Similarly to knowledge, the findings on predictors for patients' reported behaviour support the theoretical model developed. Reported behaviour is not always influenced by the same range of variables, meaning that behavioural actions result from a combination of different factors that ultimately lead to an action.

### Limitations and future perspectives

The results of this study should be considered in the light of several strengths and limitations. The major limitation of this study is the low response-rate, in all 4 participating countries. As a consequence, results should be considered with care and conclusions should be drawn with caution, as the results might not be representative of the whole driving population that takes driving-impairing medicines. However, the actual number of questionnaires that was handed out to patients was not known but the authors believe it was smaller than 3607, meaning that the actual response rate could be higher than 18%. There could be, as well, a potential selection bias. To ensure that only data referring to patients who met the inclusion criteria were part of the analysis, the survey included questions covering the inclusion criteria. Nevertheless, the authors are confident that it is possible to draw relevant conclusions.

Main results were achieved based on patient self-report of medicine use, and knowledge, attitude and behaviour related with medicines and driving. Self-report is a widely and valuable method in social sciences research [26]. Therefore, the authors trust that the information stated by patients concerning consumption of medicines is in line with pharmacy dispensing records [15] and the same stands true for self-report behaviour that is known to be an accurate proxy for observed behaviour [27]. With the present study, it was possible to determine which variables and to what extent they influence patient knowledge and reported behaviour towards medicines that influence driving ability in 4 European countries. In addition, given the fact that especially the explained variance for knowledge is quite low (10.6%), other variables, such as health literacy, history of car accidents, or ethnicity may play an important role in patient knowledge and reported behaviour and should be considered in further research.

To our knowledge, only one American study [15] addressed the problem of knowledge about medicines that can impair driving. However, the study conducted by MacLennan *et al*. was very much oriented to the knowledge that older adults who face serious driving safety and mobility issues have. The present study attempts to reflect knowledge and reported behaviour of a much broader and heterogeneous population, which makes it relevant for the field of medicines and driving and traffic safety. By accessing predictors for patient knowledge and reported behaviour, it is possible to develop more effective campaigns that point out exactly what is relevant for the patients, i.e. campaigns tailored to the patients' needs. At the same time, it is possible to give positive feedback to patients who already possess good knowledge (and therefore do not need to change) and also to help patients' with making decisions of whether they should drive their car or not while taking driving-impairing medicines.

Future research should not only focus on information campaigns for patients but also for healthcare providers. Interventions that might raise awareness about the topic and might improve communication with patients about the risk of driving under the influence of psychotropic medicines are important topics for future research, as well. As current information does not effectively contribute to patient knowledge, the authors believe there is the need for more effective ways to communicate information to patients, which might be capable of increasing patient knowledge and changing patient attitudes towards driving under the influence of medicines. New strategies to enhance communication could make use of pictograms on the box of medicines that are known to impair driving fitness. Well designed pictograms are known to improve comprehension and recall of information and can be used to trigger the discussion between healthcare providers and patients. This could potentially contribute to increasing the patient's knowledge about the use of medicines that might affect driving fitness.

Finally, research can also contribute to develop, implement, and evaluate guidelines and other information materials and/or tools that aim to provide decision support to physicians and pharmacists in prescribing and dispensing potentially impairing medicines. Additionally, the information provided to patients during the consultation can be improved if healthcare providers know how to advise their patients, which leads to the conclusion that the advice to the patient should be part of the developed information materials as well. Without a doubt, this is also a task for implementers or policy makers.

## Conclusions

Patient knowledge regarding driving under the influence of medicines does not predict changes in driving behaviour. Rather, negative attitudes towards driving under the influence of medicines is a better predictor of changes in patients' driving behaviour.

Patients' knowledge is influenced by socio-demographic parameters such as age and educational level. Behavioural changes can be explained both in terms of changes in frequency of driving and in terms of changes in the use of driving-impairing medicines. Patients who experienced side effects and who have negative attitudes towards driving under the influence of impairing medicines are more prone to change their driving behaviour (positive influence in behaviour) than those who use motor vehicles on a daily basis or those who use anti-allergic medicines. Patients who experienced side effects and who have a good knowledge of risks of having road accidents seem to change the use of driving-impairing medicines more easily than those who come from the Netherlands or take antidepressant medicines.

Future research should focus on more effective ways to increase patient knowledge and to help patients' decision making towards driving behaviour and medication intake. This could be done by implementing new strategies of communication in order to prevent patients from driving under the influence of medicines. Special attention should be paid to healthcare providers as they are the main source of information for patients. By increasing healthcare provider awareness about medicines and driving, we believe that patient knowledge will also increase and as a consequence could be responsible for a decrease in patients' driving frequency or a stabilisation in patient behaviour regarding changes in the use of medicines.

## Competing interests

The authors of this study (except for LvD) are part of the DRUID (Driving Under the Influence of Drugs, alcohol and medicines) project. DRUID is financed by the European Community within the framework of the EU 6th Framework Program (Contract No TREN-05-FP6TR-S07.61320-518404-DRUID). This article reflects only the authors' views. The European Community is not liable for any use that may be made of the information contained therein. The authors declare that they do not have any competing interests.

## Authors' contributions

SPM performed the data analysis, helped by LvD who gave substantial contributions for the statistical analysis, and drafted the final manuscript. AGV (Belgium), FJA (Spain), MH (Germany) and JJdeG (the Netherlands) designed and were responsible for the implementation of the study in the different countries. All authors read, reviewed and approved the final manuscript.

This study was financed by the European DRUID project.

## Pre-publication history

The pre-publication history for this paper can be accessed here:

http://www.biomedcentral.com/1471-2458/12/59/prepub

## Supplementary Material

Additional file 1 **Questionnaire for patients**. The use of medicines in traffic. Click here for file
