# Supplementary material for: Predictors for patient knowledge and reported behaviour regarding driving under the influence of medicines: a multi-country survey
Source: BMC Public Health. 2012 Jan 20;12:59. doi: 10.1186/1471-2458-12-59 (PMC3298461; doi:10.1186/1471-2458-12-59)
Supplement: Additional file 1 — Questionnaire for patients. The use of medicines in traffic. [file 1471-2458-12-59-S1.DOC]

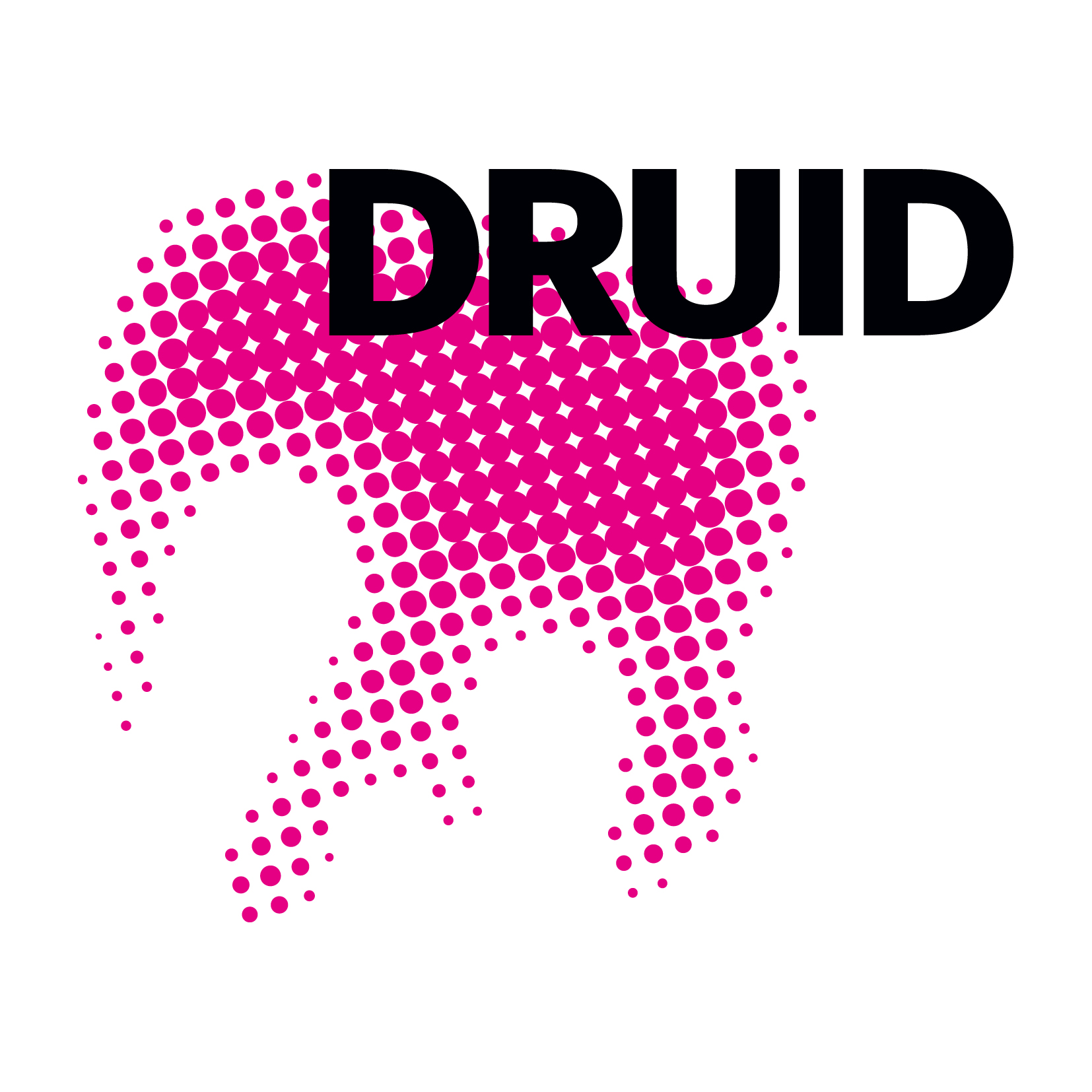


# Questionnaire for patients

# THE USE OF MEDICINES IN TRAFFIC

**DRUID task 7.4**

**Version 2. 2007.03.07**

| **Introduction to the questionnaire** |
| --- |

With the aid of this questionnaire we would like to evaluate people's opinion and knowledge about the use of potentially dangerous medicines in relation to participation in traffic. In this questionnaire you will find some questions about your participation in traffic, your use of medication and your knowledge about the influence medicines may have on the ability to drive.

Some of the questions may seem to not be applicable to your situation. For example if you are not a regular participant in traffic. Nevertheless, all the information you provide to us will be of great importance to our research. Therefore we kindly ask you to answer all questions.

The majority of the questions can be answered by simply ticking the box next to the option of your choice. In some other questions you will be asked to write down the answer yourself or to specify your choice. This questionnaire is all about your opinion and experience. So there are no correct or wrong answers.

We greatly appreciate your willingness to complete this questionnaire. After completing please return the questionnaire to <<NAME OF RESEARCH INSTITUTE>> using the stamped response envelope.

If you have any questions or remarks please feel free to call <<NAME OF CONTACT PERSON>> at <<PHONE NUMBER (and email address (optional)) OF CONTACT PERSON (optional: days and hours available for this)>>.

| **Pharmacy** |
| --- |

Firstly we would like to ask you which pharmacy supplied you with this questionnaire (we will refer to this pharmacy as “your pharmacy” or “your pharmacist” in some of our questions). The only purpose of this question is for us to determine the number of returned questionnaires per pharmacy. Privacy is guaranteed, it will not be possible to retrieve any of your personal information through this.

<<Name>>of your pharmacy:

Date of today: … … - … … - … … … … (day - month - year)

| **General information** |
| --- |

**1**. What is you gender? ❑ male

❑ female

**2.** What is your age? ……… years

**3.** What is your level of education?

- Not completed primary education
- Completed primary education
- Lower vocational training or general education
- Intermediate vocational training or intermediate and higher general education
- Higher vocational training, college or university

**4.** What is your current living status?

❑ Single, without children

❑ Single, with children

❑ (Married) Couple, without children

❑ (Married) Couple, with children

❑ Other, please specify

**5.** Please indicate the country of birth for yourself, your mother and your father.

| **Country of birth of:** | **you** | **your mother** | **your father** |
| --- | --- | --- | --- |
| Netherlands | ❑ | ❑ | ❑ |
| Indonesia / former Dutch East Indies | ❑ | ❑ | ❑ |
| Suriname | ❑ | ❑ | ❑ |
| Morocco | ❑ | ❑ | ❑ |
| Turkey | ❑ | ❑ | ❑ |
| Germany | ❑ | ❑ | ❑ |
| Netherlands Antilles | ❑ | ❑ | ❑ |
| Aruba | ❑ | ❑ | ❑ |
| Other, please specify | ……………… | ……………… | ……………… |

| **Your participation in traffic** |
| --- |

**6.** Which of the following means of transport are present in your household? *(please select all options that apply)*

❑ Bicycle

❑ Moped

❑ Motor cycle

- Car
- Bus or mini bus
- Lorry, truck or (mini)van

❑ Other, please specify:

**7.** Please indicate how often you use the following modes of transportation as a driver. *If you travelled by bicycle, moped, motor cycle, car, lorry, truck or van only if you were the driver.*

|  | 5 - 7 days per week | 2 - 4 times per week | 2 - 4 times per month | 1 time per month or less | never |
| --- | --- | --- | --- | --- | --- |
| a. Bicycle | ❑ | ❑ | ❑ | ❑ | ❑ |
| b. Moped | ❑ | ❑ | ❑ | ❑ | ❑ |
| c. Motor cycle | ❑ | ❑ | ❑ | ❑ | ❑ |
| d. Car | ❑ | ❑ | ❑ | ❑ | ❑ |
| e. Bus or mini bus | ❑ | ❑ | ❑ | ❑ | ❑ |
| f. Lorry, truck or (mini)van | ❑ | ❑ | ❑ | ❑ | ❑ |
| g. Public transport | ❑ | ❑ | ❑ | ❑ | ❑ |
| 1. Other, please specify   …………………………………… | ❑ | ❑ | ❑ | ❑ | ❑ |

**8.** Which of the following driving licenses do you have? *(please select all options that apply)*

❑ I don’t have a driving license

❑ <<I have a learner’s permit>> or << I am currently taking driving lessons>>

❑ I have a licence for a moped (category A-limited)

❑ I have a licence for a motorcycle (category A or A1)

❑ I have a licence for a car (category B, or B-E)

❑ I have a licence for a bus or mini bus (category D, D1, D-E or D1-E)

❑ I have a licence for a lorry, truck or (mini) van (category C, C1, C-E or C1-E)

❑ Other, please specify:

**9.** Do any of the members of your household, other than yourself, have a driving license?

❑ No

- Yes

**10.** Are you currently enrolled in a general education course (e.g. school, college, university) ?

❑ No → PLEASE GO TO QUESTION 12

❑ Yes

**11**. How do you travel to your education institute? Please select **all** types of transport used.

For example if you ride a bicycle to the train station then take a train and finally walk to work then select the options by bicycle, by public transport and on foot.

❑ On foot

❑ I get a lift

❑ By bicycle

❑ By moped

❑ By motor cycle

❑ By car

❑ By public transport

❑ Other, please specify …………………………………………………………………………

**12.** Do you have a job or are you involved in any unpaid work? *(please select all options that apply)*

❑ No → PLEASE GO TO QUESTION 14

❑ Yes, paid employment

❑ Yes, unpaid work

**13.** How do you travel to work (paid or unpaid)? Please select **all** types of transport used.

For example if you ride a bicycle to the train station then take a train and finally walk to work then select the options by bicycle, by public transport and on foot.

❑ On foot

❑ I get a lift

❑ By bicycle

❑ By moped

❑ By motor cycle

❑ By car, commuting only

❑ By car, for trade or profession as: *(please select the option that applies to you)*

❑ lorry or truck driver

❑ bus driver

❑ taxi driver

❑ private driver

❑ courier

❑ other, please specify

❑ By public transport

❑ Other, please specify

**14.** Which type of transport do you use for your general daily activities (other than work or education)? *For example bringing your children to school, getting your groceries, going to social activities. (please select all options that apply)*

❑ On foot

❑ I get a lift

❑ By bicycle

❑ By moped

❑ By motor cycle

❑ By car

❑ By public transport

❑ Other, please specify ………………………………………………………………………

| If you do not use a moped, motor cycle or car for your work, education course or other activities → PLEASE GO TO QUESTION 18 |
| --- |

**15**. How much time do you usually spend travelling on an average working day (all modes of transportation included)?

❑ Less than 15 minutes

❑ Between 15 and 30 minutes

❑ Between 30 and 60 minutes

❑ Between 60 and 90 minutes

❑ More than 90 minutes

**16.** If you would travel by public transport instead of using your own transport how much extra time would that take you every day? *(please estimate)*

❑ Using public transport is quicker

❑ It takes the same amount of time

❑ Up to 15 minutes extra

❑ Up to 30 minutes extra

❑ Up to 60 minutes extra

❑ More than 60 minutes extra

**17.** Would you be willing to use public transport instead of using your own transport?

❑ Yes

❑ No

If not, why? ………………………………………………………………………………

………………………………………………………………………………………

| **Medicines in traffic** |
| --- |

Questions 18 - 22 are about the use of medicines regarding the ability to drive. When you do not know the answer to any of these questions, please indicate so. This is also of great importance to this research.

**18.** Do you know that certain medicines may have a negative effect on the ability to drive?

❑ No → PLEASE GO TO QUESTION 21

❑ Yes

**19.** If yes, what kind of medicines do you think could have this effect?

Please take your time to think this over

……………………………….……………………………………………………………………

……………………………….……………………………………………………………………

……………………………….……………………………………………………………………

**20.** And which kind of negative effects do you think these medicines could have?

Please take your time to think this over

……………………………….……………………………………………………………………

……………………………….……………………………………………………………………

……………………………….……………………………………………………………………

***21.*** *How often do you think that the factors mentioned below are (part of) the cause of road accidents?* (please select one option only for every factor)

|  | **never** | **seldom** | **sometimes** | **often** | **don't know** |
| --- | --- | --- | --- | --- | --- |
| - Driving when tired | ❑ | ❑ | ❑ | ❑ | ❑ |
| - Driving under the influence of alcohol | ❑ | ❑ | ❑ | ❑ | ❑ |
| - Too short a distance to leading car | ❑ | ❑ | ❑ | ❑ | ❑ |
| - Speeding | ❑ | ❑ | ❑ | ❑ | ❑ |
| - Use of medicines that might impair driving | ❑ | ❑ | ❑ | ❑ | ❑ |
| - Use of illicit drugs | ❑ | ❑ | ❑ | ❑ | ❑ |
| - Use of a mobile phone while driving | ❑ | ❑ | ❑ | ❑ | ❑ |

**22.** To which extent do you agree or disagree on the following statements?

*(please select one option per statement*)

|  | **totally agree** | **agree** | **disagree** | **totally disagree** | **no opinion** |
| --- | --- | --- | --- | --- | --- |
| - - 1. The risk of having a road accident is smaller when you have just started taking a driving impairing medicine compared to long term treatment | ❑ | ❑ | ❑ | ❑ | ❑ |
| - - 1. The risk of having a road accident is similar when you take more of a driving impairing medicine than prescribed | ❑ | ❑ | ❑ | ❑ | ❑ |
| - - 1. The risk of having a road accident may increase when you combine a driving impairing medicine and over the counter medicines (e.g. pain killers, cough remedy) | ❑ | ❑ | ❑ | ❑ | ❑ |
| - - 1. The risk of having a road accident increases when you use alcohol while taking a driving impairing medicine | ❑ | ❑ | ❑ | ❑ | ❑ |
| - - 1. The risk of having a road accident remains the same when you use several driving impairing medicines at the same time | ❑ | ❑ | ❑ | ❑ | ❑ |
| - - 1. The risk of having a road accident increases with a high dose of a driving impairing medicine | ❑ | ❑ | ❑ | ❑ | ❑ |

| **Use of medicines** |
| --- |

**23.** Do you use any of the following prescribed medicines? (please indicate all options that apply)

❑ Sedatives

❑ Tranquillizers

❑ Medicines for depression

❑ Medicines for allergies

❑ No → PLEASE GO TO QUESTION 38

**24.** Please fill in the table below **only for your sedatives or tranquillizers or medicines for depression and/or for allergies?** Please take into account only the medicines that you actually take. We kindly ask you to state the dosage you use, how many times a day and at which times for each medicine. Please record since when you have been using these medicines as well. In case you use more than three of these medicines please record those you have been using for the longest period of time. Please try to fill in the table as completely as you can.

| **Name of medicine**  Please copy this directly from the label or package | **dosage per tablet / capsule?** | **how many tablets/capsules do you take at these moments of the day?** | | | | **since when?** (month/ year) |
| --- | --- | --- | --- | --- | --- | --- |
|  |  | **morning** | **afternoon** | **evening** | **night** |  |
| 1. | …… mg | …… | …… | …… | …… | ... / …… |
| 2. | …… mg | …… | …… | …… | ……. | ... / …… |
| 3. | …… mg | …… | …… | …… | …… | ... / …… |

**25.** Please indicate which of the following side effects you experience or have experienced while using these medicines? *(please select all options that apply)*

❑ Sleepiness or drowsiness

❑ Decreased alertness

❑ Problems concentrating

❑ Blurred view

❑ Dizziness

❑ I did not experience any side effects

❑ Other, please specify

**26.** Thinking of the last time your General Practitioner (GP) or specialist prescribed you the above mentioned sedative, tranquillizer or medicine for depression or for allergy. Please indicate which of the following actions he/she carried out during this visit?

| The GP/specialist has… | **no** | **not really** | **more or less** | **yes** |
| --- | --- | --- | --- | --- |
| - - 1. … discussed several options of treatment with me. | ❑ | ❑ | ❑ | ❑ |
| - - 1. … consulted me about the choice of medicine he/she prescribed (related to possible side effects). | ❑ | ❑ | ❑ | ❑ |
| - - 1. …asked me for my daily activities (e.g. driver by trade, handling heavy machinery) before he/she prescribed the medicine. | ❑ | ❑ | ❑ | ❑ |
| - - 1. … involved me in decisions about the support or treatment I receive. | ❑ | ❑ | ❑ | ❑ |
| e. … asked me whether I drive/ride a motorised vehicle prior to prescribing the medicine. | ❑ | ❑ | ❑ | ❑ |

| Information about medicines |
| --- |

Questions 27 - 32 cover the information you **received** about your medicines.

**27.** Did you at any time receive information regarding the possible influence of one of your medicines on your ability to drive? *(please select all options that apply)*

❑ No → PLEASE GO TO QUESTION 33

❑ Yes, I spontaneously received information from the GP/specialist or pharmacist.

❑ Yes, after I asked my GP/specialist or pharmacist for the information myself.

**28**. Which medicine(s) did this concern?

**29.** Who informed you about the possible influence of this medicine on your ability to drive? And how did you receive this information? *(please select all that apply)*

|  | **GP, specialist or other doctor** | **pharmacist** | **I was not informed** |
| --- | --- | --- | --- |
| 1. Oral information | ❑ | ❑ | ❑ |
| 1. Written information (e.g. brochure) | ❑ | ❑ | ❑ |
| 1. With reference to the product information in the box | ❑ | ❑ | ❑ |
| 1. With reference to the warning label on the box | ❑ | ❑ | ❑ |
| 1. With reference to the general label on the box | ❑ | ❑ | ❑ |
| 1. Other, please specify | ❑ | ❑ | ❑ |

**30.** What kind of information did you receive? *(please select all options that apply)*

❑ To which extent the medicine influences the ability to drive

❑ How long the medicine continues to affect the ability to drive

❑ Availability of alternative medicines for your disease/illness causing less impairment of the ability to drive

❑ Other, please specify

If you received information about the possible influence on the ability to drive for more than one of your medicines please answer questions 31 and 32, thinking of the last time you received information..

**31.** Did the information you received change your frequency of driving?

❑ No, because:

❑ I did not think the information was relevant to me

❑ It was not feasible for me to change my frequency of driving

❑ I did not notice any negative effects that influence my driving ability

❑ other, please specify

❑ Yes, and:

❑ I decided *not* to drive a motorised vehicle anymore

❑ I decided to drive/ride a motorised vehicle *less often*

❑ I decided to drive/ride a motorised vehicle on *less parts of the day*

❑ other, please specify

**32.** Did the information you received change anything about your use of the driving impairing medicine?

❑ No, because:

❑ I did not think the information was relevant to me

❑ there was no alternative medicine available

❑ other, please specify

❑ Yes, and:

❑ I decided *not* to use the medicine

❑ I decided to use (most of) the medicine at night instead of during the day

❑ I decided to only use the medicine when I did not need to be driving

❑ I asked for or I was prescribed a medicine causing less impairment of the ability to drive

❑ other, please specify

| Questions 33 - 37 cover the information about your medicines you **searched for** yourself |
| --- |

**33.** Did you look for any information regarding the possible influence on your ability to drive caused by any of your medicines?

❑ No → PLEASE GO TO QUESTION 38

❑ Yes

**34.** Where did you look for this information? *(please select all options that apply)*

❑ <<I have contacted the national medicines information phone number>>

❑ I have looked in a medical reference book

❑ I have searched in magazines

❑ I have searched the following internet pages: *(please select the ones you have visited)*

❑ the medicines manufacturer’s internet page

❑ an internet page about my disease or illness

❑ an internet page about medicines, for example <<[www.apotheek.nl](http://www.apotheek.nl/)>>

❑ a general internet page about one’s health

❑ a health care insurance company’s internet page

❑ (an)other internet address(es), please specify

❑ Other, please specify

**35.** Did you find the information you were looking for?

❑ No → PLEASE GO TO QUESTION 38

❑ Yes

If so, where did you find it?

**36.** Did the information you found change your frequency of driving?

❑ No, because:

❑ I did not think the information was relevant to me

❑ It was not feasible for me to change my frequency of driving

❑ I did not notice any negative effects that influence my driving ability and thus frequency of driving

❑ I found information stating the medicine does not have any driving impairing effects

❑ Other, please specify………………………………………………………………..

❑ Yes, and:

❑ I decided *not* to drive a motorised vehicle anymore

❑ I decided to drive/ride a motorised vehicle *less often*

❑ I decided to drive/ride a motorised vehicle on *less parts of the day*

❑ Other, please specify………………………………………………………………..

**37.** Did the information you found change your use of this driving impairing medicine?

❑ No, because:

❑ I did not think the information was relevant to me

❑ there was no alternative medicine available

❑ other, please specify

❑ Yes, and:

❑ I decided *not* to use the medicine

❑ I decided to use (most of) the medicine at night instead of during the day

❑ I decided to only use the medicine when I did not need to be driving

❑ I asked for or I was prescribed a medicine causing less impairment of the ability to drive

❑ other, please specify

| Questions 38 and 39 cover the patient information leaflet in the medicine box. |
| --- |

**38.** Do you usually read the patient information leaflet?

❑ Yes, I read the entire patient information leaflet

❑ Yes, I read certain sections of the patient information leaflet, being:

*(please select all sections you read)*

❑ section “what kind of medicine is this and what is it being used for”

❑ section “what you should know before you start using/taking this medicine”

❑ section “ how is this medicine used/taken”

❑ section “possible adverse effects of the medicine”

❑ section “how to store the medicine”

❑ No, I do not read the product information

**39.** How clear is the information in the medicine’s product information to your opinion?

❑ Very clear

❑ Fairly clear

❑ Not very clear

❑ Not clear at all

**40.a.** Information about medicines can be provided by several sources or institutes. Whom would you preferably receive information from concerning driving impairing medicines?

*(please select one* option only)

| Information about … | **from GP/**  **specialist** | | **from pharmacist** | **from govern-ment** | **from medicine’s manufacturer** | **no preference** |
| --- | --- | --- | --- | --- | --- | --- |
| a .…the medicine’s possible influence on driving ability | | ❑ | ❑ | ❑ | ❑ | ❑ |
| b …how long the medicine continues to affect the ability to drive | | ❑ | ❑ | ❑ | ❑ | ❑ |
| c … availability of alternatives for this medicine causing less impairment of the ability to drive | | ❑ | ❑ | ❑ | ❑ | ❑ |

**40.b.** Information about medicines can be provided via several ways. How would you preferably receive this information?

*(please select one option only)*

❑ Orally

❑ Written

❑ Pictograms on the medicines package

❑ Other, please specify

| Attitude towards behaviour in traffic |
| --- |

Questions 41 and 42 ask for **your opinion** regarding some statements about behaviour in traffic.

**41.** To which extent do you agree or disagree on the following statements?

(please select one option only)

|  | **totally agree** | **agree** | **disagree** | **totally disagree** | **no opinion** |
| --- | --- | --- | --- | --- | --- |
| 1. When using driving impairing medicines people should decide for themselves whether they drive/ride a motorised vehicle or not | ❑ | ❑ | ❑ | ❑ | ❑ |
| 1. Driving while using driving impairing medicines should be punished more severely in the future | ❑ | ❑ | ❑ | ❑ | ❑ |
| 1. Driving after the consumption of alcohol should be prohibited | ❑ | ❑ | ❑ | ❑ | ❑ |
| 1. The risk of driving under the influence of driving impairing medicines is being exaggerated | ❑ | ❑ | ❑ | ❑ | ❑ |
| 1. The risks of driving under the influence of alcohol are being exaggerated | ❑ | ❑ | ❑ | ❑ | ❑ |

**42.** To which extent do you agree or disagree on the following statements?

*(please select one option only)*

|  | **totally agree** | **agree** | **disagree** | **totally disagree** | **no opinion** |
| --- | --- | --- | --- | --- | --- |
| 1. Possible consequences of the use medication in traffic have never crossed my mind | ❑ | ❑ | ❑ | ❑ | ❑ |
| 1. When I drive when using a driving impairing medicine I endanger my personal safety | ❑ | ❑ | ❑ | ❑ | ❑ |
| 1. When I drive when using a driving impairing medicine I endanger the safety of other traffic participants | ❑ | ❑ | ❑ | ❑ | ❑ |
| 1. If I know someone is using driving impairing medicines I will not let them drive me | ❑ | ❑ | ❑ | ❑ | ❑ |
| 1. When I have been prescribed a driving impairing medicine I choose not to use my car and choose other types of transportation | ❑ | ❑ | ❑ | ❑ | ❑ |
| 1. I do not mind other traffic participants using driving impairing medicines | ❑ | ❑ | ❑ | ❑ | ❑ |
| 1. When I have been prescribed a driving impairing medicine I try to use my car/vehicle as little as possible | ❑ | ❑ | ❑ | ❑ | ❑ |
| 1. When other drivers participate in traffic they take their use of driving impairing medicines into account | ❑ | ❑ | ❑ | ❑ | ❑ |

| Remarks |
| --- |

Do you have any remarks as a result of this questionnaire? Please express them here.

| Thank you very much for completing this questionnaire! |
| --- |

Please send the questionnaire to <<NAME OF RESEARCH INSTITUTE>> using the enclosed stamped addressed envelope.
